# THE 4MS IN PRIMARY CARE: THE GERIATRIC INTERPROFESSIONAL TEAM TRANSFORMATION IN PRIMARY CARE (GITT-PC) MODEL

**DOI:** 10.1093/geroni/igz038.2830

**Published:** 2019-11-08

**Authors:** Daniel Moran

**Affiliations:** Dartmouth-Hitchcock Medical Center, Lebanon, New Hampshire, United States

## Abstract

GITT-PC is a Dartmouth GWEP developed program that builds on lessons learned from the original GITT program funded by the John A. Hartford Foundation in the 1990s. GITT-PC improves delivery of healthcare to older adults in primary care by training healthcare professionals in team functioning, rapid cycle QI, and evidence based geriatric practice. The program capitalizes on the role of nursing and other healthcare disciplines. To maximize sustainability, it focuses on Medicare-reimbursable visits, including: the Annual Wellness Visit (AWV), Chronic Care Management (CCM), Advance Care Planning (ACP), and Transitional Care Management (TCM). The model’s standardized approach to implementation begins with practice assessments and two trainings. Practices participate in a data-driven, virtual learning collaborative with monthly data collection and learning sessions. Since 2015, GITT-PC has been implemented in 14 sites in northern New England, 10 sites in upstate New York, and nationally through five other GWEP awardees across the country.

